# p53 Interacts with RNA Polymerase II through Its Core Domain and Impairs Pol II Processivity *In Vivo*

**DOI:** 10.1371/journal.pone.0022183

**Published:** 2011-08-04

**Authors:** Sunyoung Kim, Sri Kripa Balakrishnan, David S. Gross

**Affiliations:** Department of Biochemistry and Molecular Biology, Louisiana State University Health Sciences Center, Shreveport, Louisiana, United States of America; J. David Gladstone Institutes, and the University of California San Francisco, United States of America

## Abstract

The tumor suppressor p53 principally functions as a gene-specific transcription factor. p53 triggers a variety of anti-proliferative programs by activating or repressing the transcription of effector genes in response to genotoxic stress. To date, much effort has been placed on understanding p53's ability to affect transcription in the context of its DNA-binding activity. How p53 regulates transcriptional output independent of DNA binding is less well understood. Here we provide evidence that human p53 can physically interact with the large subunit of RNA polymerase II (Pol II) both in *in vitro* interaction assays and in whole cell extracts, and that this interaction is mediated (at least in part) through p53's core DNA-binding domain and the Ser5-phosphorylated CTD of Pol II. Ectopic expression of p53, combined with mutations in transcription elongation factors or exposure to drugs that inhibit Pol II elongation, elicit sickness or lethality in yeast cells. These phenotypes are suppressed by oncogenic point mutations within p53's core domain. The growth phenotypes raise the possibility that p53 impairs Pol II elongation. Consistent with this, a p53-dependent increase in Pol II density is seen at constitutively expressed genes without a concomitant increase in transcript accumulation. Additionally, p53-expressing yeast strains exhibit reduced transcriptional processivity at an episomal reporter gene; this inhibitory activity is abolished by a core domain point mutation. Our results suggest a novel mechanism by which p53 can regulate gene transcription, and a new biological function for its core domain that is susceptible to inactivation by oncogenic point mutations.

## Introduction

p53 is a tumor suppressor that transcriptionally regulates upwards of 2500 genes in response to genotoxic stress. In so doing, it triggers a variety of anti-proliferative programs [1]. Through its sequence-specific binding, p53 can promote transcription of genes involved in cell cycle arrest, apoptosis, and DNA repair. Targeted differently, it can repress transcription of genes involved in survival, G2/M transition, and metastasis [2], [3]. p53 contains four principal functional domains: an N-terminal transactivation domain, a core DNA binding domain, a tetramerization domain and a C-terminal regulatory domain [4]. At the N-terminus, there are two highly acidic transactivation (TA) domains, TA1 (amino acids 1–40) and TA2 (amino acids 40–83) [5], [6]. Each domain has distinct interacting partners and exhibits independent transactivation activity [7]. Together they mediate p53's interaction with several general transcription factors (GTFs) including TBP, TAF1, TFIIB and TFIIH to stimulate gene transcription [8], [9]. The tetramerization domain (amino acids 325–356) mediates intermolecular formation of four p53 monomers to form a tightly packed tetramer, which is biologically active and efficiently binds to p53 response elements (PREs). The regulatory domain (amino acids 363–393) exhibits extensive post-translational modification [10] and has been implicated in regulating both p53's transactivation and DNA binding activities [11], [12].

Because of p53's critical role in tumor suppression, mutations within the p53 gene are found in nearly half of all human cancers [13]. 97% of these are missense mutations that map to the core domain (amino acids 102–292), a region that plays a key role in mediating p53's tumor suppressive activity [14], [15]. The core domain not only exhibits sequence-specific DNA binding activity within the nucleus, it also has a cytoplasmic role in which it activates Bax, leading to permeabilization of the outer mitochondrial membrane and initiation of the apoptotic cascade [16].

The core domain is composed of a common immunoglobulin scaffold with a DNA-binding surface formed by a loop–sheet–helix (LSH) motif and two β-turn loops (L2 and L3) tethered by a single zinc atom [17]. Six “hot spot” mutations map to the core domain and these mutations are divided into two classes depending on their effect on protein folding and DNA-binding ability [18]. Contact mutations such as R248W (L3) and R273H (ß-sheet 10 [S10] in LSH) abolish p53's ability to bind specific DNA sequences and activate expression of its target genes. Conformational mutations occur in amino acids required for maintenance of p53 structure and disrupt the structural stability of p53. Examples include R175H (L2), G245D (L3), R249S (L3), and V143A (ß-sandwich) [14], [19]. These mutations can reduce the binding activity of p53 to its DNA sequence, specific peptides, and cellular and viral proteins [20], [21]. Therefore, mutations in the core domain of p53 can affect cell fate not only by regulating DNA binding activity, but also by interfering with p53's protein interactions.

Previous work has shown that p53's interactions with GTFs and co-activators are crucial to promote or inhibit target gene transcription. While much focus has been placed on p53 as an activator of transcription, less is known of the mechanisms by which p53 represses transcription. Indeed, significant numbers of genes are down-regulated in a p53-dependent manner. This repression mechanism appears to be more diverse than p53-mediated transcription activation [22]. Proposed mechanisms include (i) inhibiting the assembly of the PIC and (ii) remodeling chromatin structure by recruitment of histone deacetylases (HDACs) and other enzymatic complexes [9]. Importantly, it has been suggested that binding of p53 to its DNA sequence is not necessary for its inhibitory activity, although the presence of a functionally active core domain is required [9], [23]. In addition, several studies have raised the possibility that p53 is involved in regulating transcription elongation as a consequence of the cross-talk between events at the promoter and those regulating elongation [24]. Indeed, p53 can interact with proteins that are involved in elongation. An example of this is TFIIH [25], which has been shown to be involved not only in initiation but also in promoter escape [26]. The Pol II elongation factor ELL has also been reported as a physical and functional interacting partner of p53 [27]. Interestingly, this physical association leads to suppression of *p21* transcription. Likewise, hPAF1C, an elongation factor, directly interacts with p53 both *in vitro* and *in vivo* (cited in [28]).

In this study, we extend our earlier analysis suggesting that p53 can physically interact with Pol II, both *in vivo* and *in vitro* [29] . We provide evidence that p53's physical and functional interaction with Pol II requires integrity of its core domain, and that expression of p53 in the model eukaryote *Saccharomyces cerevisiae* impairs Pol II elongation at both chromosomal and episomal genes. Oncogenic mutations within the p53 core domain obviate this effect. These and other results suggest a novel mechanism by which p53 can regulate gene transcription in eukaryotic cells.

## Results

### p53 preferentially binds to peptides that mimic the phosphorylated state of the Pol II CTD

**Figure 1:**
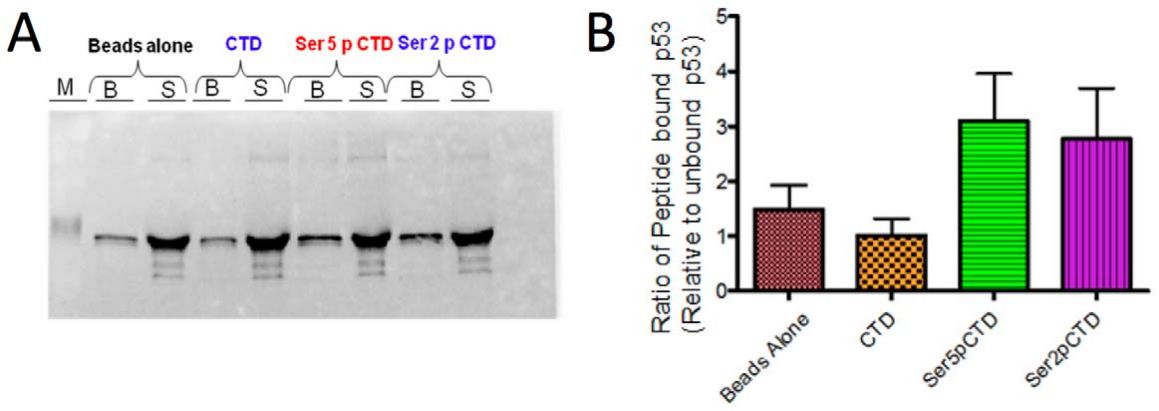
p53 Binds to Peptides that Mimic the Phosphorylated Pol II CTD. Biotinylated CTD peptides bearing four heptad repeats (28-mers) that were unphosphorylated (CTD), Ser5-phosphorylated (Ser5-P-CTD), or Ser2-phosphorylated (Ser2-P-CTD), were adsorbed to streptavidin-coated magnetic beads. Recombinant HA-p53, bearing an HA tag on its N-terminus, was incubated with beads alone or beads bearing peptides. Bound proteins (B) and those remaining in the supernatant (S) were subjected to SDS-PAGE and the presence of p53 was detected using the 1801 mAb. (B) Graphical summary of peptide-bound p53 relative to unbound p53. Depicted are means ± S.E.M. (N = 4). A two-sample *t*-test (two-tailed, equal variance) comparing p53 binding to the Ser5-phosphorylated CTD peptide *vs.* the unphosphorylated CTD peptide results in a *P* value of 0.058. A corresponding analysis of p53 binding to the Ser2-phosphorylated CTD peptide results in a *P* value of 0.11.

Previous work from our laboratory has suggested that p53 may regulate Pol II elongation through a novel mechanism that involves its physical interaction with the Pol II large subunit, Rpb1 [29]. To explore this possibility further, we investigated whether p53 can bind the phosphorylated C-terminal domain (CTD) of Rpb1. We employed synthetic peptides mimicking the CTD, which consists of heptad repeats of the evolutionarily conserved sequence, YSPTSPS (26 repeats in yeast, 52 in human), and acts as a platform for the binding of regulatory factors during transcription [30]. As elongating RNA polymerase is characterized by phosphorylation at Ser5 and/or Ser2 within the heptad repeat, we tested p53 affinity for synthetic peptides bearing four perfect heptad repeats that were unmodified or phosphorylated at one or the other residue. Unphosphorylated, Ser5-phosphorylated or Ser2-phosphorylated peptides biotinylated on their N-terminus were incubated with recombinant human p53. Bound and unbound fractions were subjected to immunoblot analysis, and peptide-bound p53 was quantified as the ratio of bound p53 to unbound p53. As shown in Figure 1 (panels A and B), human p53 preferentially binds Ser5-phosphorylated CTD peptides (*P* = 0.058), and to a lesser degree Ser2- phosphorylated CTD peptides (*P* = 0.11). It shows no affinity for the unphosphorylated peptide over beads alone. Taken together with observations that p53 and Rpb1 co-purify from yeast whole cell extracts (WCEs) (see below), these assays suggest that p53 can physically interact with the Pol II large subunit through its phosphorylated CTD (see Discussion).

### The core domain of p53 is required for the p53-Rpb1 interaction

**Figure 2:**
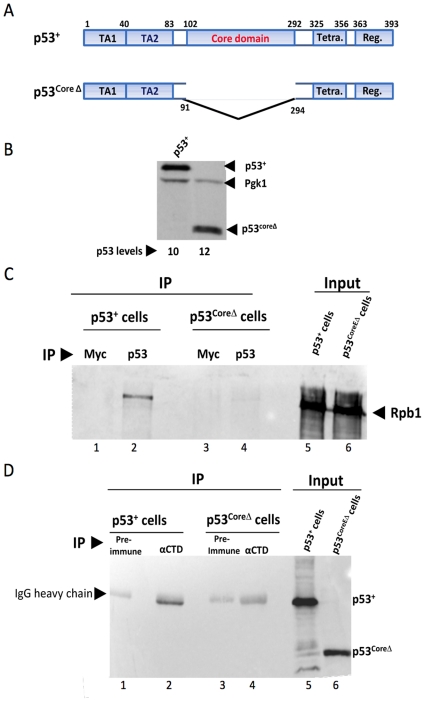
The Core Domain of p53 Mediates the p53-Pol II Interaction in Whole Cell Extracts. (A) Domain structures of p53^+^ and p53^coreΔ^. TA1 and TA2, transactivation domains 1 and 2; Tetra., tetramerization domain; Reg., regulatory domain. (B) p53 immunoblot analysis of cells expressing p53^+^ (strain SY1000) and p53^coreΔ^ (strain SY1004) regulated by the *GAL1* promoter. Cells were grown in rich medium containing 1.8% raffinose and 0.2% galactose. p53 was detected through use of the DO-1 mAb; expression levels were internally normalized to those of Pgk1. (C) The Pol II large subunit co-immunoprecipitates with p53^+^, but not with p53^coreΔ^, in yeast WCEs. Immunoprecipitates were obtained from WCEs isolated from strains SY1000 and SY1004 cultivated as above using Myc- or p53-specific antibodies (9E10 or DO-1, respectively) (lanes 1–4). Rpb1 was detected using anti-CTD antiserum. Lanes 1 and 3 serve as negative controls as cells do not express a Myc-tagged protein. (D) p53^+^, but not p53^coreΔ^, co-IPs with Pol II. Immunoprecipitates were generated using either pre-immune or anti-Pol II (CTD) antiserum (lane 1–4) from extracts as above. p53 and p53^coreΔ^ were detected using DO-1.

Physical association of p53 with the Pol II large subunit raises the question of which domain of p53 is responsible for mediating this interaction. It has been previously shown that the presence of the core DNA-binding domain is required for p53-dependent repression, although binding of p53 to PREs is unnecessary [9], [23]. Based on this, we hypothesized that the core domain might mediate p53's interaction with Pol II, and performed a reciprocal co-immunoprecipitation (co-IP) comparing full-length p53 (p53^+^) with core domain-deleted p53 (p53^coreΔ^). Both p53^+^ and p53^coreΔ^ (Figure 2A) were expressed from integrated, *GAL1*-regulated genes. Immunoblot analysis shows that p53^coreΔ^ is expressed at levels similar to that of full-length p53 in cells grown in the presence of 0.2% galactose/1.8% raffinose (Figure 2B). As observed previously [29], Rpb1 co-immunoprecipitates with full-length p53 from a yeast WCE using the p53-specific monoclonal antibody (mAb) DO-1 (Figure 2C, lane 2). In important contrast, DO-1 fails to co-immunoprecipitate Rpb1 from a WCE prepared from yeast expressing p53^coreΔ^ (Figure 2C, lane 4), indistinguishable from the negative controls (lanes 1, 3; cells are Myc^−^). Likewise, a polyclonal anti-Pol II (Rpb1) antibody fails to co-IP p53^coreΔ^ in the reciprocal reaction, even though it efficiently co-IPs p53^+^ (Figure 2D, compare lane 4 vs. 2). These results indicate that the core domain is necessary for the p53-Pol II interaction in whole cell extracts.

### Core domain point mutations suppress p53-mediated synthetic growth defects

**Figure 3:**
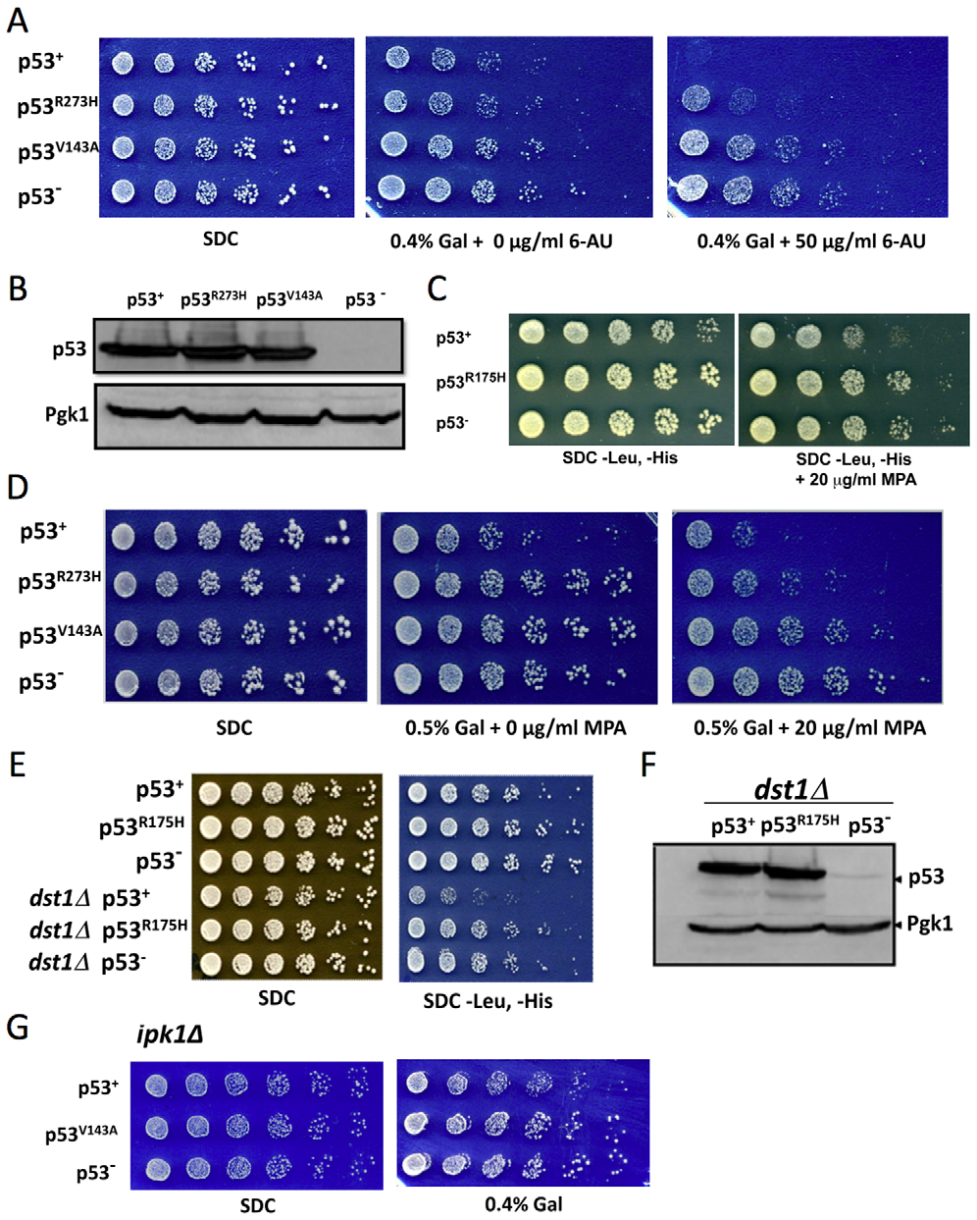
Core domain Mutations Abolish Synthetic Growth Phenotypes Observed in p53-Expressing Cells. (A) Fivefold serial dilutions of yeast cells (BY4741 background) expressing p53^+^, p53^R273H^ or p53^V143A^ behind the *GAL1* promoter or expressing no ectopic protein (p53^−^). Cells were spotted onto synthetic rich medium containing either 2% glucose (SDC) or 1.6% raffinose/0.4% galactose; 6-azauracil (6-AU) was added where indicated. Cells were incubated at 30°C for 3 to 6 days. (B) Immunoblot analysis of p53^+^ and p53 core domain mutant-expressing strains grown in liquid synthetic medium containing 1.6% raffinose/0.4% galactose. p53^+^ and its mutant derivatives were detected using the 1801 mAb. (C) As in A, except p53 expression was regulated by the *ADH1* promoter and BY4741 cells were grown on synthetic rich medium containing 2% glucose in the absence or presence of mycophenolic acid (MPA). (D) Spot dilution analysis of p53^+^ and p53 mutant-expressing cells as in A except grown on synthetic rich medium containing either 2% glucose or 1.5% raffinose/0.5% galactose in the absence or presence of MPA. (E) Fivefold serial dilutions of strain BY4741 (rows 1–3) or its isogenic *dst1Δ* counterpart (rows 4–6) transformed with p53^+^- or p53^R175H^-expressing plasmids (or empty vector; p53^−^) as in C and spotted onto synthetic rich medium. The SDC panel, while non-selective, shows that roughly equivalent numbers of cells were spotted. Note that in replicate experiments, p53^+^ BY4741 cells exhibited a slow growth phenotype on SDC -Leu, -His similar to that seen in C. (F) Immunoblot analysis of p53^+^ and p53^R175H^ expression in the *dst1Δ* mutant grown in synthetic rich medium containing 2% glucose. (G) Spot dilution analysis of p53^+^ and p53^V143A^-expressing BY4741 *ipk1Δ* cells grown on synthetic medium containing either 2% glucose (SDC) or 1.6% raffinose/0.4% galactose as indicated.

p53-expressing cells exhibit elevated sensitivity to the anti-elongation drug, 6-azauracil (6-AU), and synthetic growth phenotypes in combination with mutations in transcription elongation factors [29]. To test the involvement of the core domain, we asked whether oncogenic point mutations localized within this domain would impact these phenotypes. We tested both contact (R273H) and conformational (V143A, R175H) point mutations. As illustrated in Figure 3 (panels A and D), expression of moderate levels of p53^+^, comparable to what is seen in activated human cell lines [29], is mildly toxic (compare growth of p53^+^ vs. p53^−^ cells on 0.4% galactose-containing medium). When combined with an inhibitor of Pol II elongation – either 6-AU or mycophenolic acid (MPA) [31] – p53^+^ causes sickness or lethality. Strikingly, both V143A and R175H conformational point mutations suppress these phenotypes (Figure 3, panels A, C and D; note that p53 expression in panel C is under control of the constitutive *ADH1* promoter). And although less robust, suppression is also conferred by the R273H point mutation (panels A, D). These suppressing effects are unlikely to stem from instability of either mutant protein, since intracellular levels of p53^+^, p53^R273H^ and p53^V143A^ are essentially equivalent (panel B; see also panel F).

We next asked whether loss of core domain function alleviates the severe slow growth phenotype of p53^+^ cells depleted of the elongation factor TFIIS (Dst1) [29]. TFIIS facilitates Pol II elongation by enhancing the intrinsic 3′-end cleavage and backtracking activity of Pol II following its stalling or arrest. In this assay (Figure 3E), p53 derivatives were under control of the *ADH1* promoter and cells were spotted on either non-selectable medium (SDC; serves as a load control) or selectable medium (SDC -Leu, -His). Strikingly, the slow growth phenotype of p53^+^ *dst1Δ* cells is entirely alleviated by the R175H point mutation (compare row 5 with rows 4, 6). Once again, this suppression cannot be accounted for by instability (or diminished expression) of the mutated protein (Figure 3F).

Our observations that synthetic growth defects arise when p53 is combined with either an elongation inhibitor or depletion of TFIIS are consistent with the possibility that p53 negatively regulates Pol II elongation. Nonetheless, it is conceivable that these synthetic phenotypes, particularly those obtained through use of drugs, have little to do with Pol II elongation. To address this concern, we asked whether a mutation in a gene whose physiological function is unrelated to Pol II elongation elicits comparable synthetic growth defects when combined with expression of p53^+^. For this purpose we selected a mutation, *ipk1*Δ, that was previously shown to elicit strong genetic interactions when combined with 6-AU [32]. As Ipk1 encodes a nuclear protein required for synthesis of 1,2,3,4,5,6-hexakisphosphate, it is unlikely that its deletion directly affects Pol II elongation. When combined with *ipk1*Δ, moderate p53^+^ expression fails to trigger synthetic sickness beyond that observed in the WT parent BY4741 strain (Figure 3G; compare to Figure 3A), contrasting significantly with the synthetic sickness of the p53^+^ *dst1*Δ strain (Figure 3E). Taken together, the genetic interaction data are consistent with a model in which p53 impedes Pol II elongation, and that missense oncogenic mutations within the core domain suppress this activity.

### p53 influences Pol II density at constitutively transcribed genes

**Figure 4:**
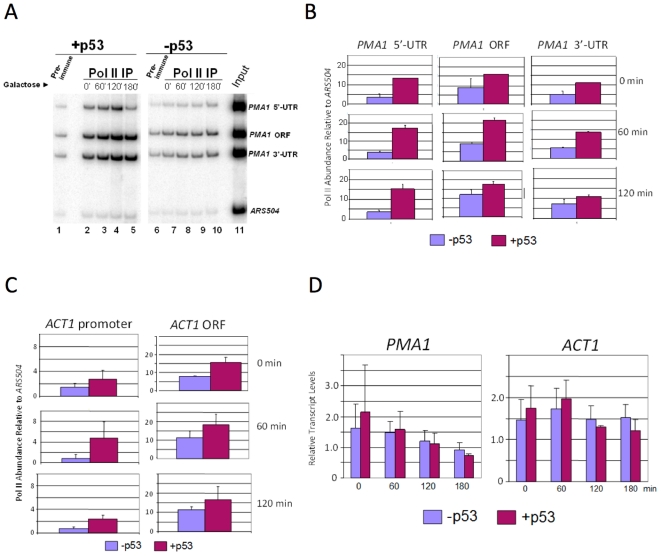
p53 Increases Pol II Density at Constitutively Transcribed Genes. (A) Pol II ChIP analysis of the *PMA1* gene in p53^+^ and p53^−^ cells. Depicted is a multiplex PCR analysis (resolved on an 8% polyacrylamide gel) of DNA purified from immunoprecipitated chromatin samples isolated from SY1000 (*P_GAL1-_ p53*) and SLY101 (p53^−^) cells subjected to a 2% galactose induction for 0, 60, 120 and 180 min. Immunoprecipitations were conducted using pre-immune (lanes 1, 6) or anti-CTD antiserum (lane 2–5, 7–10). Lane 11, DNA isolated from chromatin used in the ChIPs of lanes 1 and 2. An ORF-free region on chromosome V (*ARS504*) was co-amplified with the *PMA1* loci, and serves as a non-specific IP control. (B) Summary of Pol II ChIP assays of the *PMA1* 5′-UTR, ORF, and 3′-UTR in p53^+^ and p53^−^ cells conducted as in panel A. Depicted is Pol II abundance at each locus (net signal, immune minus pre-immune) relative to *ARS504* (net signal, immune minus pre-immune). Shown are means ± S.D; N = 2. (C) As in panel B, except Pol II ChIP analysis of the *ACT1* gene. (D) Northern analysis of *PMA1* and *ACT1* in p53^+^ and p53^−^ cells following addition of galactose for the indicated times. Transcript levels were normalized to those of *SCR1* and are presented in arbitrary units (shown are means ±S.D; N = 2).

We next asked whether p53's association with polymerase influenced the density of Pol II within gene coding regions. This might be anticipated if p53 impacted Pol II processivity (stalling), elongation rate, or both. As shown in Figure 4, p53 increased Pol II density within the *PMA1* 5′-UTR, ORF and 3′-UTR (Figure 4A, compare p53-expressing [lanes 2–5] vs. p53-non-expressing cells [lanes 7–10]; quantified in Figure 4B). Pol II density is also increased within the *ACT1* promoter and ORF (Figure 4C). Increased Pol II density is most pronounced at the 5′-UTR/promoter of both genes. Interestingly, the p53-dependent effect is evident in cells that have not been induced by galactose and express p53 at a constitutively low level (∼10% the level present in induced cells [29]). Increasing intracellular p53 levels does not cause a further increase in Pol II density at either gene, although the reason for this is unclear. To test whether increased Pol II is correlated with the transcription of either *PMA1* or *ACT1*, we performed northern blot analysis. Transcript levels of these two genes were not significantly affected by increased Pol II abundance in p53-expressing cells (Figure 4D). This result implies that increased Pol II density stems from impaired elongation rate. Output is not affected since the rate-limiting step for these two genes, as suggested by the data, is apparently at initiation. These observations are consistent with the idea that p53's association with Pol II diminishes its elongation efficiency.

### p53 impairs Pol II processivity at episomal PHO5/lacZ genes in a core domain-dependent manner

**Figure 5:**
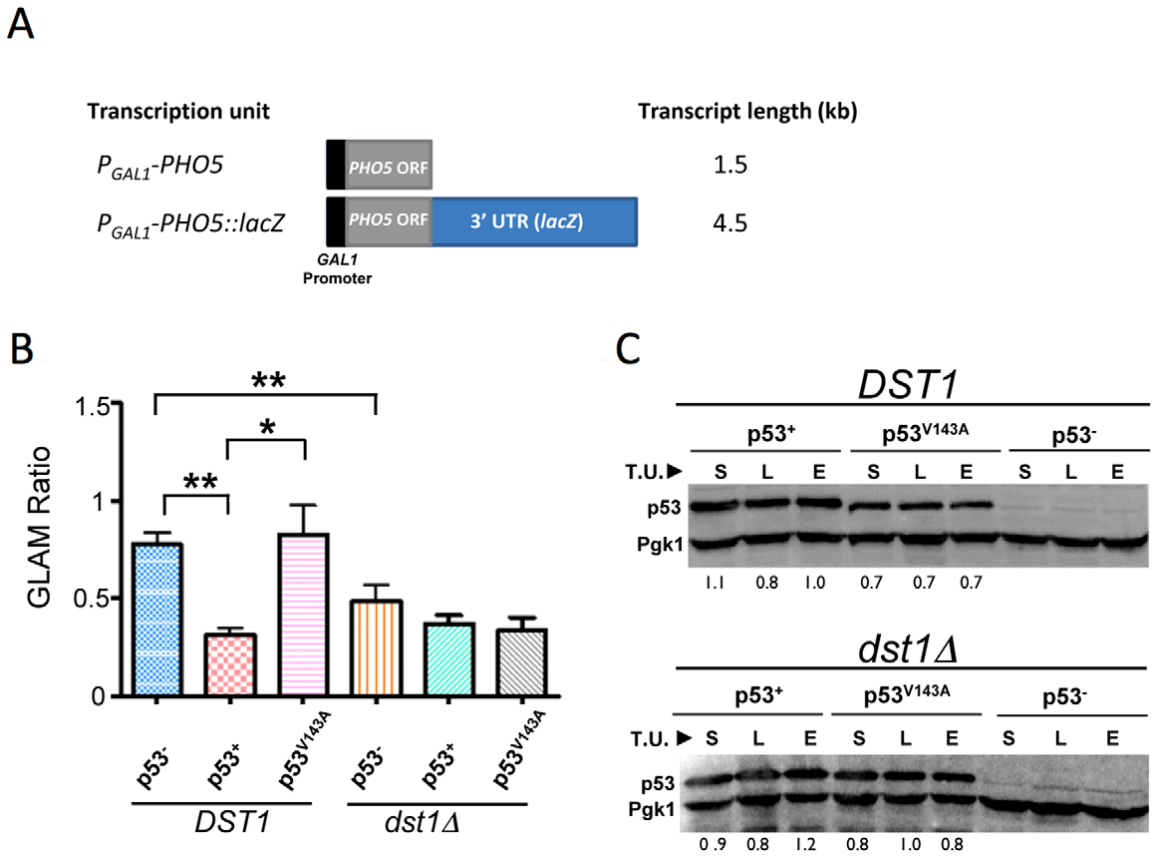
p53^+^, but not the Oncogenic Mutant p53^V143A^ , Impairs Pol II Processivity on the Episomal *PHO5-lacZ* Gene. (A) Physical maps of *PHO5* reporter genes under *GAL1* regulation used in the processivity assay. (B) Isogenic *DST1^+^* and *dst1*Δ strains (SLY101 background) expressing the indicated p53 derivatives were transformed with either *P_GAL_-PHO5* or *P_GAL_*-*PHO5::lacZ* reporter plasmids, and grown in synthetic selectable medium containing 1% raffinose and 1% galactose to early log phase. Cells were harvested and *PHO5* expression levels were inferred by an acid phosphatase assay. GLAM ratio refers to the acid phosphatase activity of cells expressing the long transcript (*PHO5-lacZ*) relative to those expressing the short transcript (*PHO5*). Depicted are mean values ± S.E.M. of six independent transformants of each indicated strain/plasmid combination. Single asterisk signifies a significant difference between the indicated values (*P*<0.01; two-tailed *t* test); double asterisk signifies *P*<0.0005. (C) Western blot analysis of p53^+^ and p53^V143A^ levels in *DST1^+^* and *dst1*Δ strains employed in the GLAM assay (panel B). p53 derivatives were detected using the 1801 mAb; their relative levels, normalized to those of Pgk1, are provided below each lane. S, short transcript expressing cells; L, long transcript plasmid transformed cells; E, cells transformed with empty vector; T.U., transcription unit.

The forgoing observations and previous results [29] raise the possibility that p53 perturbs transcription elongation. To test this idea further, we performed an *in vivo* transcription elongation assay that was developed for detection and analysis of factors involved in transcription elongation [33]. This assay is based on comparison of Pho5 acid phosphatase expression arising from two transcription units sharing the same promoter, ORF, and terminator but differing in the length of their 3′-untranslated regions (3′-UTR) (illustrated in Figure 5A). Mutations in elongation factors cause a greater reduction in the steady-state levels of long versus short transcript (termed the GLAM ratio), and this can be conveniently monitored by measuring acid phosphatase activity [33].

Plasmids bearing either short or long transcription units (Figure 5A) were transformed into isogenic p53^+^ and p53^−^ strains, and the GLAM ratio of p53^+^ cells was compared to that of p53^−^ cells. In p53^−^ cells, expression of long versus short transcription units was fairly similar (GLAM ratio ∼0.8; Figure 5B). In contrast, p53^+^ cells exhibit nearly a 60% reduction in phosphatase activity of long transcript-expressing cells relative to short transcript-expressing cells. This deficiency in mRNA biogenesis is comparable to cells lacking TFIIS (*dst1Δ*), and is consistent with the idea that p53 can suppress Pol II processivity. It is also consistent with observations reported above that the p53-induced increase in Pol II density at *PMA1* and *ACT1* is most pronounced within the 5′-end of these genes (Figure 4, panels B and C). We next investigated if the core domain plays a role in this process by testing whether the V143A conformational mutation could alleviate this reduction. As shown in Figure 5B, the V143A missense mutation restored the GLAM ratio to a level indistinguishable from that seen in p53^−^ cells. To rule out the possibility that the V143A effect arose from diminished p53 levels, we performed immunoblot analysis. This assay revealed that p53^V143A^ levels were comparable to those of p53^+^ (Figure 5C, upper panel, lanes 4–6 vs. 1–3). Finally, to test whether p53 elicits a synergistic effect when combined with *dst1Δ*, we measured the GLAM ratio of *dst1Δ* cells expressing p53^+^. However, we observed only a slight decrease in GLAM ratio beyond that elicited by the *dst1Δ* mutation alone (Figure 5B) despite a high level of p53^+^ expression (Fig. 5C, lower panel). While the reason for this is unclear (see Discussion), the GLAM data suggest that p53 can impede Pol II processivity in a model eukaryotic system, and that integrity of the core domain of p53 is required for this activity.

## Discussion

We demonstrate in this study that the p53 tumor suppressor can physically and functionally interact with the large subunit of RNA polymerase II. This physical interaction involves the core DNA-binding domain of p53 and, at minimum, the Ser5-phosphorylated C-terminal domain of Rpb1. Short phosphopeptides mimicking the Ser5-phosphorylated CTD (and to a lesser degree, the Ser2-phosphorylated CTD) bind recombinant human p53 in an *in vitro* pull-down assay. Given that human Rpb1 has 52 heptad repeats, the intrinsic affinity of p53 for the phosphorylated CTD is likely to be much higher than we observed with biotinylated peptides containing four repeats; thus the data reported in Figure 1 may understate the difference in p53's affinity for the phosphorylated *vs.* unphosphophorylated CTD. Interaction of p53 with the phosphorylated CTD is consistent with our previous work which demonstrated that the Ser5 phosphorylated Pol II subunit co-purifies with p53 from yeast WCEs [29]. Physical interaction of a gene-specific transcription factor with the Pol II CTD is unusual, but not unprecedented. It has been shown in at least one other case, that of the HIV regulatory protein Tat [34]. Tat's interaction with the CTD appears to affect CTD phosphorylation by CDK2/cyclin E.

Several lines of evidence suggest that the core DNA-binding domain of p53 is required for its physical and function interaction with Pol II. First, a p53 mutant lacking amino acids 91–294, termed p53^coreΔ^, fails to co-purify with the Pol II large subunit from yeast WCEs using either p53- or Rpb1-specific antibodies. This is in contrast to full-length p53, which co-immunoprecipitates with Rpb1 using either antibody. Second, oncogenic point mutations within the core domain, particularly those that disrupt its tertiary structure (V143A and R175H), suppress the synthetic growth phenotypes of p53-expressing yeast depleted of elongation factors TFIIS or Isw1 (Figure 3 and data not shown), or of yeast cultivated in the presence of the anti-elongation drugs 6-AU or MPA. Third, the V143A conformational mutation suppresses p53-mediated reduction of Pho5 phosphatase encoded by a long transcript, suggesting that the core domain is important for the reduction in Pol II processivity seen in p53^+^ cells.

Notably, p53 regulates transcription in yeast cells independent of its DNA-binding activity. This follows from our previous work [29] as well as from the fact that none of the genes evaluated here – *PMA1*, *ACT1* and *PHO5/lacZ* – bear consensus p53-response elements, either within their promoters or coding regions. Given this, we propose the following model. p53, through its core domain, physically interacts with the Ser5-phosphorylated CTD of elongating Pol II. By a mechanism that is as yet unclear, p53 negatively affects Pol II processivity. One way that it could do this is by displacing elongation factors bound to the transcription elongation complex (TEC). An appealing candidate is TFIIS, which binds Pol II cooperatively with the elongation factor Paf1C [28], and whose depletion strongly impairs Pol II processivity at the episomal *PHO5::lacZ* gene (this study and [33]). Consistent with this idea, p53 elicits as strong an effect on Pol II elongation at *PHO5::lacZ* as does depletion of TFIIS. Moreover, the combination of p53 and *dst1Δ* elicits no more severe effect on Pol II processivity than either perturbation alone. Clearly, this cannot be a general mechanism by which p53 acts in this model system, given the strong synthetic growth phenotype of a p53^+^ *dst1Δ* strain. For at least one essential gene, p53 must be acting in a different pathway than TFIIS, hence the synergy seen when the two insults are combined.

A major consideration of this work is whether the novel activity of p53 identified in yeast is conserved in higher eukaryotes. In fact, several studies have shown a direct interaction between p53 and human elongation factors such as TFIIH, ELL and hPAF1C [25], [27], [28]. This raises the possibility that p53's negative effect on transcription elongation is conserved from yeast to humans. It is likely that p53's interaction with the TEC will be highly regulated in mammalian cells. As guardian of the genome, p53 might exhibit this activity to suppress expression of pro-proliferative genes in response to genotoxic stress. Mammalian cells contain much larger, intron-rich genes. Thus, the rather subtle elongation defects elicited by p53 on the representative yeast genes studied here, particularly with respect to transcriptional output, may significantly underestimate its impact on the much longer genes of higher eukaryotes. Complementing our results, Hatakeyama's lab has shown that p53 can inhibit transcription elongation by targeting ELL, an elongation factor that increases the rate of Pol II transcription by suppressing transient pausing [27]. Interestingly, through use of ChIP, we have observed a low level of p53 association with the coding regions of several oncogenes in human RKO and MCF7 cell lines in response to genotoxic stress (D. Gross, R. Beckerman, A. Barsotti and C. Prives, unpublished observations). While the biological significance of this observation is unclear, it is consistent with the premise that p53 can negatively affect the expression of pro-proliferative genes through a mechanism that involves its physical association with the Pol II transcription elongation complex.

## Materials and Methods

### Strain Construction

Strains used in this study are listed in Table 1. To construct a strain expressing p53 deleted of its core domain (amino acids 91–294) (p53^CoreΔ^) from an integrated expression cassette, we used a modification of the cloning-free PCR-based allele replacement strategy of Rothstein and colleagues [35] essentially as described previously [29]. Strain YYO2 was used as recipient and genomic DNA isolated from SY1000, harboring an integrated *P_GAL1_-p53* gene at the *LEU2* locus, was used as template. *LEU2 -P_GAL1_-p53(codons 1–90)* was fused to *p53(codons 295–393) - LEU2* through use of a 24 nt adaptamer homologous to codons 295–302. The resultant fusion was targeted to the *leu2-3,112* locus of YYO2 by homologous recombination, and the *P_GAL1_-p53* ^CoreΔ^ integrant confirmed by genomic PCR analysis and DNA sequencing. For all PCR reactions, amplifications were performed using Phusion High Fidelity DNA Polymerase (New England Biolabs). *GAL1*-regulated centromeric p53 expression vectors (Yp53, p53^R273H^ and p53^V143H^) were generously provided by Bert Vogelstein. *ADH1*-regulated centromeric expression vectors pRB16 and p53^R273H^ were generously provided by Rainer Brachmann and Carol Prives, respectively.

**Table 1:** Yeast Strains.

Strain	Genotype	Source or reference
SLY101	*MATα ade^−^ can1-100 cyh2^r^ his3-11,15 leu2-3,112 trp1-1 ura3*	[40]
YYO2	SLY101; *p53-hsp82*	[29]
SY1000	YYO2; *leu2-3::P_GAL1_-p53-LEU2*	[29]
SY1004	YYO2; *leu2-3::P_GAL1_-p53^Core^* ^Δ^ *-LEU2*	This study
BY4741	*MATa his3Δ1 leu2Δ0 met15Δ0 ura3Δ0*	Research Genetics
YDR007W	BY4741; *trp1*Δ	Research Genetics
K1000	YDR007W; pRS316 (*URA3 CEN6*), pRS314 (*TRP1- CEN6*)	This study
K1001	YDR007W; Yp53 (*P_GAL1_-p53 URA3 -CEN6*), pRS314	This study
K1002	YDR007W; p53 ^R273H^ (*P_GAL1_-p53 ^R273H^ TRP1-CEN6*), pRS316	This study
K1003	YDR007W; p53 ^V143A^ (*P_GAL1_-p53 ^V143A^ TRP1-CEN6*), pRS316	This study
K1004	BY4741; pRS313 (*HIS3 -CEN6*), pRS315 (*LEU2- CEN6*)	This study
K1005	BY4741; pRB16 (*P_ADH1_-p53 HIS3 -CEN6*), pRS315	This study
K1006	BY4741; p53 ^R175H^ (*P_ADH1_-p53 ^R175H^ LEU2-CEN6*), pRS313	This study
YGL043W	BY4741; *dst1Δ*, pRS313, pRS315	Research Genetics
YGL043W1	YGL043W; pRB16, pRS315	This study
YGL043W2	YGL043W; p53 ^R175H^, pRS313	This study
YDR315C	BY4741; *ipk1*Δ	Research Genetics
YDR315C1	BY4741; *ipk1*Δ, pRS316	This study
YDR315C2	BY4741; *ipk1*Δ, Yp53	This study
YDR315C3	BY4741; *ipk1*Δ, p53-1^V143A^ (*P_GAL1_-p53 ^V143A^ URA3-CEN6*)	This study
G1000	SLY101; pSCh202 (*P_GAL1_-PHO5 URA3 CEN6*), pRS314	This study
G1001	SLY101; pSCh212 (*P_GAL1_-PHO5 URA3 CEN6*), pRS314	This study
G1002	SLY101; pSCh202, Yp53	This study
G1003	SLY101; pSCh212, Yp53	This study
G1004	SLY101; pSCh202, Yp53^V143A^	This study
DCY103	SLY101; *dst1*Δ	[29]
D1000	DCY103; pSCh202, pRS314	This study
D1001	DCY103; pSCh212 , pRS314	This study
D1002	DCY103; pSCh202, Yp53	This study
D1003	DCY103; pSCh212, Yp53	This study
D1004	DCY103; pSCh202, Yp53 ^V143A^	This study
D1005	DCY103; pSCh212, Yp53 ^V143A^	This study

### Genetic Assays

For spot dilution assays, BY4741 was transformed with plasmids encoding wild-type (WT) p53, p53 core domain mutants, or vector alone and pre-grown to stationary phase in the appropriate selectable medium. Cultures were then diluted to OD_600_ 0.5 and transferred to a 96-well microtiter dish. Each was then fivefold serial diluted using double distilled water and applied to solid synthetic medium containing 2% glucose, 1.6% raffinose/0.4% galactose, or 1.5% raffinose/0.5% galactose with or without elongation inhibitory drugs (6-AU or MPA). Cells were grown at 30°C for 3∼6 days to allow formation of visible colonies.

### Western Blot Analysis

Isolation of WCEs was conducted essentially as described [36]. WCEs were separated on 10% SDS-polyacrylamide gels and electroblotted onto nitrocellulose membranes (Bio-Rad). Blotted membranes were incubated with antibodies specific for p53 (DO-1 [Santa Cruz Biotechnology, Inc.] or 1801 [hybridoma supernatants generated at Cold Spring Harbor Laboratory; gift of Carol Prives]) or Pgk1 (Molecular Probes) and then incubated with horseradish peroxidase-conjugated anti-mouse or anti-rabbit secondary antibodies. Protein bands were visualized by using ECL Plus Western blotting detection reagents (GE Healthcare) and detected and quantified on a Storm 860 PhosphorImager utilizing Image Quant TL v.2003.02 software.

### Co-Immunoprecipitation Assay

Co-immunoprecipitation was performed as described previously [29], with yeast strains SY1000 and SY1004 grown to early log phase in rich media containing 1.8% raffinose and 0.2% galactose. The following antibodies and amounts were used for each IP: [i] p53 (DO-1; Santa Cruz), 2 µg; [ii] Myc (9E10; Santa Cruz), 2 µg; and [iii] Pol II CTD (rabbit antiserum raised against a GST-mouse CTD fusion protein bearing 52 heptad repeats [37]), 3 µl.

### GLAM Assay

SLY101 and its *dst1*Δ counterpart were transformed with plasmids expressing p53^+^ or p53 ^V143A^, or with vector alone (pRS316). These transformants were then transformed with either short transcript plasmid (pSCh202, containing *P_GAL_-PHO5*) or long transcript plasmid (pSCh212, containing *P_GAL_-PHO5::lacZ*) (kindly provided by Daniel Ginsburg and Sebastian Chavez), and grown to OD_600_ 0.3–0.7 in synthetic medium lacking both tryptophan and uracil and containing 1% galactose and 1% raffinose. Cells were collected by centrifugation and acid phosphatase activity was assayed as described [33].

### CTD Pull-Down Assay

A modified CTD pull-down assay [38] was performed. Briefly, 0.5 mg of biotinylated peptides (comprised of four CTD heptad repeats; Anaspec) were bound to 50 µl of streptavidin-coated dynabeads (Invitrogen) by incubation in the presence of 100 µl of high salt binding buffer (25 mM Tris-HCl [pH 8.0]); 1 M NaCl; 1 mM dithiothreitol; 5% glycerol; 0.03% Nonidet P-40) at 4°C for 2 hr. The peptide-bound beads were washed once with 500 µl of CTD-wash buffer (25 mM Tris-HCl [pH 8.0]; 50 mM NaCl; 1 mM dithiotheritol; 5% glycerol; 0.5 µg/µl BSA; 0.1% Triton X-100), then pre-incubated for 2 hr with 500 µl of CTD-binding buffer (same as CTD-wash buffer except contained 1 µg/µl BSA) to block non-specific binding. Peptide-bound beads were then resuspended in 100 µl of CTD binding buffer and approximately 120 ng of *E.coli* expressed HA-p53 (generous gift of O. Laptenko and C. Prives, Columbia University) were incubated at 4°C for 2 hr. The beads were collected magnetically and the supernatant saved as the unbound fraction. Beads were washed with CTD-wash buffer 3 times and 1/5 volume of each bound fraction and each unbound fraction was resuspended in 6× sample buffer and subjected to SDS-PAGE. Western blot analysis was performed using monoclonal antibody 1801.

### Chromatin Immunoprecipitation (ChIP)

ChIP was conducted essentially as described [29]. Briefly, 400 µl of soluble, crosslinked chromatin were immunoprecipitated using 3 µl CTD antiserum (described above); as the negative control, an equivalent volume of chromatin was immunoprecipitated using 3 µl pre-immune serum. DNA, purified from the IP, was dissolved in 25–30 µl distilled water; 2 µl were used as template in a multiplex PCR performed in the presence of α-^32^P-dATP. Amplified DNA was electrophoretically resolved on an 8% polyacrylamide gel using PhosphorImager. For quantification, we subtracted a representative pre-immune ChIP sample from each IP signal prior to calculating Q_locus_ ( = IP_locus_/Input_locus_). PCR primers used were as follows (all coordinates relative to ATG): *ACT1* promoter (forward, −294 to −272; reverse, −45 to −68); *ACT1* ORF (forward, +606 to +629; reverse, +1000 to +978); *PMA1* promoter (forward, −370 to −349; reverse, −51 to −72), *PMA1* coding region (forward, +1010 to +1032; reverse, +1235 to +1215); *PMA1* 3′-UTR (forward, +2018 to +2040; reverse, +2177 to +2154).

### Northern Analysis

RNA isolation, formaldehyde gel electrophoresis and blotting to nylon membranes were conducted as described previously [29]. Hybridization probes spanned the following coordinates (relative to ATG for *ACT1* and *PMA1*): *ACT1*, +606 to +1000; *PMA1*, +2742 to +2872; *SCR1*, +343 to +467.

### p53 Binding Site Search

To search for potential p53 binding sites within the coding and/or flanking regions of *ACT1*, *PMA1*, *PHO5* and *lacZ*, the “find sequence function” in Vector NTI (ver. 11.0) was used with ‘RRRCWWGYYY’ as the search string, where ‘R’ = purine, ‘Y’ = pyrimidine, and ‘W’ = A or T. The search was first conducted with the tolerance set to 0 mismatches with follow-up searches of up to 3 mismatches to detect any potential p53 binding sites, defined as a 20 bp sequence consisting of two repeats of RRRCWWWGYYY separated by 0–13 bp [39]. None were identified.
